# Li-Decorated Ti_2_CF_2_ MXene for Efficient Solid-State Hydrogen Storage

**DOI:** 10.1021/acsomega.6c02092

**Published:** 2026-04-10

**Authors:** Bilal Gülseven, Gokhan Surucu, Ozge Surucu, Aysenur Gencer

**Affiliations:** a Graduate School of Natural and Applied Sciences, 37511 Gazi University, Ankara 06500, Türkiye; b Department of Energy Systems Engineering, Faculty of Technology, 37511 Gazi University, Ankara 06500, Türkiye; c Department of Energy Systems Engineering, Faculty of Engineering and Natural Sciences, 226850 Ankara Yildirim Beyazit University, Ankara 06010, Türkiye; d Department of Physics, K. O. Faculty of Science, 166263 Karamanoglu Mehmetbey University, Karaman 70200, Türkiye

## Abstract

Efficient hydrogen storage is a major challenge for clean energy technologies. This study investigates the potential of Li-decorated Ti_2_CF_2_ MXene as a hydrogen storage material using density functional theory. Our calculations show that Li atoms bind stably to the Ti_2_CF_2_ surface. Ab initio molecular dynamics simulations confirm that the Li atoms do not cluster at room temperature due to strong electrostatic repulsion. The material adsorbs hydrogen molecules via a physisorption mechanism, which allows for reversible storage. It is found that double-sided Li decoration significantly improves the performance, achieving a gravimetric capacity of 3.81 wt % (26 H_2_ molecules). The calculated desorption temperatures indicate that hydrogen can be released under practical conditions. These findings suggest that Li-decorated Ti_2_CF_2_ is a mechanically robust and dynamically stable candidate for hydrogen storage applications, offering a balanced trade-off between binding strength and reversibility.

## Graphical abstract

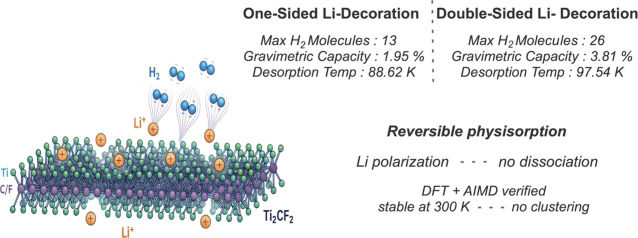

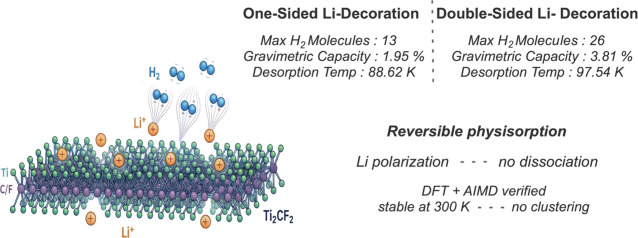

## Introduction

Today’s environmental and energy policies indicate that it is necessary to move away from hydrocarbon-based energy systems rapidly. In particular, the increasing concentration of greenhouse gases in the atmosphere and the limited reserves of fossil fuels have led to the beginning of a global scientific consensus on the urgent need for carbon purification.^1,2^ Within this paradigm, hydrogen (H_2_) has emerged not just as an alternative fuel but as an essential vector for sustainable energy economics.^2,3^

Hydrogen has the highest gravimetric energy density of 120 MJ/kg among chemical fuels, which is almost three times higher than methane,^3,4^ which has a value of 55.6 MJ/kg.^5^ In addition, H_2_ produces water as the only byproduct by its combustion or electrochemical oxidation in fuel cells, eliminating direct carbon emissions.^4,6^ However, the transition to a hydrogen economy currently holds a major hurdle due to storage, which is a critical technological bottleneck. Hydrogen gas’s low volumetric density (0.0824 kg/m^3^ in STP) requires significant compression or liquefaction to achieve practical energy densities.^7,8^ These processes carry serious energy penalties and safety risks.^4,7–9^

As a result, solid-state hydrogen storage methods have become very popular as a promising solution to address these concerns.^10,11^ The storage of hydrogen in nanostructured materials through physisorption offers the potential for high volumetric density at moderate operating conditions and reduces the risks associated with high-pressure tanks.^12,13^ In recent years, various porous materials, metal–organic frameworks (MOFs), covalent organic frameworks (COFs), and carbon nanostructures, have been extensively investigated.^10,14^ On the other hand, meeting the strict gravimetric and volumetric capacity values targeted by the U.S. Department of Energy (DOE) presents significant challenges.^15–18^ For this reason, the search for new two-dimensional (2D) materials with superior surface-to-volume ratios and tunable surface chemistries continues.^19,20^

Among the broad family of 2D materials, the MXene (transition metal carbides and nitrides) group has risen as the leading candidate class thanks to their tunable surface chemistry, metallic conductivity, and mechanical flexibility.^21–23^ Titanium-based MXenes such as Ti_2_C are very interesting for hydrogen storage because Ti is a relatively light transition metal.^24^ This key feature is an important requirement for mobile applications.

In addition, the experimental synthesis of MXenes, which is usually obtained by etching the ⟨A⟩ layer from the MAX phases using hydrofluoric acid (HF), inevitably leads to surface functionalization. The resulting material is not naked Ti_2_C, but instead terminates in functional groups such as O, OH, or F.^21,25,26^ While these terminations provide thermodynamic stability, they often pose challenges for hydrogen storage. Ti_2_CF_2_, a fluorine-terminated MXene, has a highly electronegative and chemically inert surface. This immobility leads to poor physisorption of H_2_ molecules, with binding energies generally falling below the optimal window (0.2–0.6 eV/H_2_) required for reversible storage at ambient conditions.^25,26^

In this context, to overcome the tendency of the bare F-terminated surface to bond poorly, surface modification with metal decoration has been proposed as an effective strategy.^27,28^ Due to their very low atomic mass and high electropositivity, alkali metals, especially lithium (Li), are a strong candidate for this modification.^27–30^

Decorating Ti_2_CF_2_ with Li atoms creates a unique adsorption mechanism. When Li is adsorbed to the MXene surface, significant charge transfer takes place from the Li atom to the underlying Ti_2_CF_2_ substrate.^27^ This leaves the Li atoms in a cationic state (Li^+^), which allows multiple H_2_ molecules to be adsorbed through strong electrostatic polarization.^27–29^ This mechanism not only increases the hydrogen binding energy up to the desired range but also acts as active domains that prevent hydrogen aggregation, significantly enhancing gravimetric capacity.^27,29^

In this work, the hydrogen storage performance of Li-decorated Ti_2_CF_2_ is systematically investigated using density functional theory (DFT). In particular, the stability of Li decoration was studied for the prevention of metal aggregation. In addition, gravimetric capacities were calculated to assess whether this system could meet the practical hydrogen storage targets of the U.S. Department of Energy (DOE).

### Computational Details

In this study, the Li-decorated Ti_2_CF_2_ MXene has been investigated using the Vienna ab initio simulation package (VASP)^31,32^ based on density functional theory. The electron–electron interactions have been considered using the Perdew–Burke–Ernzerhof (PBE) functional^33^ within the generalized gradient approximation (GGA), while the electron–ion interactions have been considered using the projector-augmented wave (PAW) method.^34,35^ The calculations have been performed with an energy cutoff of 530 eV, and the energy and force convergence criteria have been employed as 10^–7^ eV per unit cell and 10^–6^ eV/Å, respectively. The Ti_2_CF_2_ MXenes have been optimized using 15 × 15 × 1 gamma-centered *k*-points,^36^ while Li-decorated Ti_2_CF_2_ MXenes and H_2_-adsorbed Li-decorated Ti_2_CF_2_ MXenes have been optimized using 4 × 4 × 1 gamma-centered *k*-points.^36^ The D3 algorithm^37^ has been employed for the van der Waals correction in the calculations, while it is imperative to create a vacuum space that is 15 Å in order to prevent interactions between the layers. The Bader partial charge calculations have been executed through utilization of VASP, while the subsequent analysis has been conducted employing the algorithm devised by Henkelman et al.,^38^ which is founded upon Bader’s proposal.^39^ The elastic constants have been calculated using the energy-strain method using the ELASTOOL program.^40,41^ The determination of the H_2_ positions has been facilitated by implementing the Cap Like Initial Conditions (CLICH) algorithm.^42^ For the purposes of the present algorithm, it is assumed that the bond length of the H_2_ molecule is 0.74 Å, as established by experimental research.^43^ The values for θ, *z*_max_, and *r*_up_ are set to 45°, 3 Å, and 1 Å respectively, for *n* = 1–5. In the systems under consideration for the *n* ≥ 6, the radial distance between the H_2_ molecules is fixed at *h* = 1.3 Å, as opposed to the fixed radius (*r*_up_). Visualizations of the crystal structures and the respective electronic band structures are obtained by utilizing the VESTA program^44^ and the Sumo tools.^45^

To assess the thermal stability of the Li-decorated structures at room temperature, the ab initio molecular dynamics calculations are carried out under zero pressure. A Langevin thermostat^46,47^ is used with the following atomic frictions for Ti, C, F, and Li atoms and lattice frictions, respectively: 10, 3, 5, 3, and 4 ps^–1^. The Verlet algorithm is applied to integrate the equations of motion related to the ions with a time step of 1 fs. The AIMD calculations are both computationally expensive and time-consuming, so the machine-learned force field (MLFF) implemented in VASP^48,49^ is encouraged. The descriptors based on the Gaussian representation of atomic distributions^49,50^ are used for the description of the machine-learned potential energy surface. In addition, reliable estimates of the target properties, including energy, forces, and stress tensor components, are obtained using Bayesian linear regression.^48,51^

## Results and Discussion

### Structural and Stability Considerations for Ti_2_CF_2_ MXene

The Ti_2_CF_2_ MXenes have been obtained through a two-step process. First, aluminum atoms are removed from the Ti_2_AlC MAX phase by etching. Second, fluorine atoms are added to the surface for surface functionalization. In the present study, F atoms were chosen for surface functionalization on the basis that the most stable structure in the Computational 2D Materials Database (C2DB)^52,53^ was obtained by functionalizing with F. Figure 1 illustrates the crystal structure of the Ti_2_CF_2_ MXenes from different perspectives, and Table 1 lists the lattice parameter obtained from the optimized structure. The optimized lattice constant for the Ti_2_CF_2_ MXene is consistent with the literature as listed in Table 1. The vacuum space for this MXene is taken as 19.8 Å. Formation energy constitutes a significant parameter in determining the thermodynamic stability of a given material. The formation energy for the Ti_2_CF_2_ MXene was calculated using the equation outlined in ref 54 and is provided in Table 1. According to the data presented in Table 1, the negative value obtained for the formation energy indicates this material’s thermodynamic stability. In addition to thermodynamic stability, the mechanical stability of the material under scrutiny is determined through the calculation of its elastic constants. In the case of a hexagonal 2D lattice, the elastic constants required are referred to as *C*_11_ and *C*_12_. It is vital to ensure that these constants satisfy the Born stability criteria.^55,56^ As demonstrated in Table 1, it is evident that the criteria are both met, i.e., *C*_11_ > 0 and *C*_11_ > |*C*_12_|.^55,56^ Consequently, the material has been demonstrated to exhibit both mechanical and thermodynamic stability. The Young’s modulus represents a material’s capacity for resistance to being stretched, and the Ti;_2_CF_2_ MXene has a high Young’s modulus value. It is imperative to note that the shear modulus of a material is indicative of its resistance to shear stress; thus, the Ti_2_CF_2_ MXene is exhibiting a moderate shear modulus. The Poisson ratio is a property of a material that indicates the degree of contraction in the perpendicular in-plane direction when the material is subjected to stretching along one axis. The Ti_2_CF_2_ MXene has low Poisson’s ratio.

**Figure 1:**
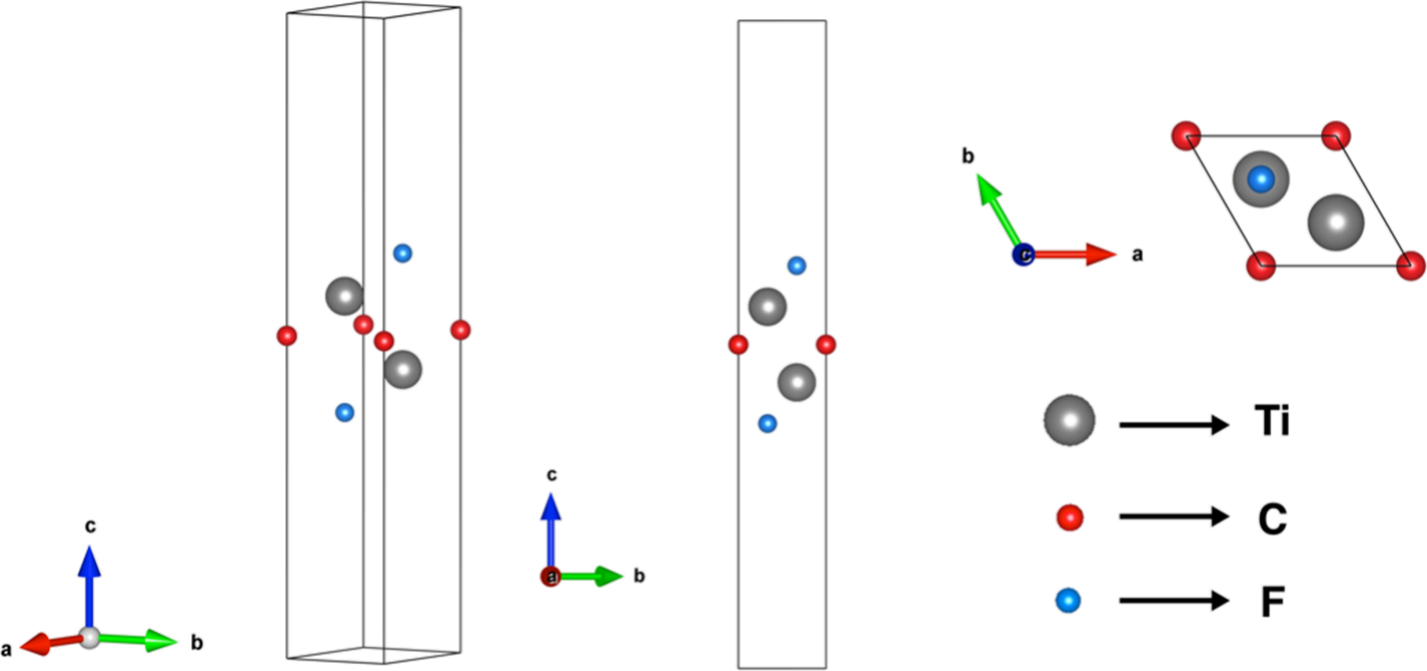
Crystal structure for the Ti_2_CF_2_ MXene from different perspectives.

**Table 1:** Optimized Lattice Parameters (*a* in Å), Formation Energy (*E*_for_ in eV/Atom), Elastic Constants (*C*_11_ and *C*_12_ in N/m), Young's Modulus (*Y*_2D_ in N/m), Shear Modulus (*G*_2D_ in N/m), and Poisson’s ratio (ν) for the Ti_2_CF_2_ MXene

*a*	*E*_for_		*C*_11_
3.10	–1.61		176.93
3.043 ^57^			
3.048 ^58^			
3.063 ^59^			

*C*_12_	*Y*_2D_	*G*_2D_	ν
52.10	161.59	62.42	0.29

The dynamical stability of the Ti_2_CF_2_ MXene has been the subject of evaluation by means of the linear response method using a 2 × 2 × 1 supercell. As evidenced by Figure 2, the dynamic stability of the Ti_2_CF_2_ MXene is characterized by a lack of imaginary frequencies in its phonon dispersion curves. Furthermore, the phonon density of states (DOS) is examined, while projections are made onto atomic species. Projections indicate that C atoms contribute predominantly to high-frequency vibrational modes, while Ti and F atoms are predominantly present in the low- and midfrequency ranges. The distribution under consideration is indicative of the lighter atomic mass of C.

**Figure 2:**
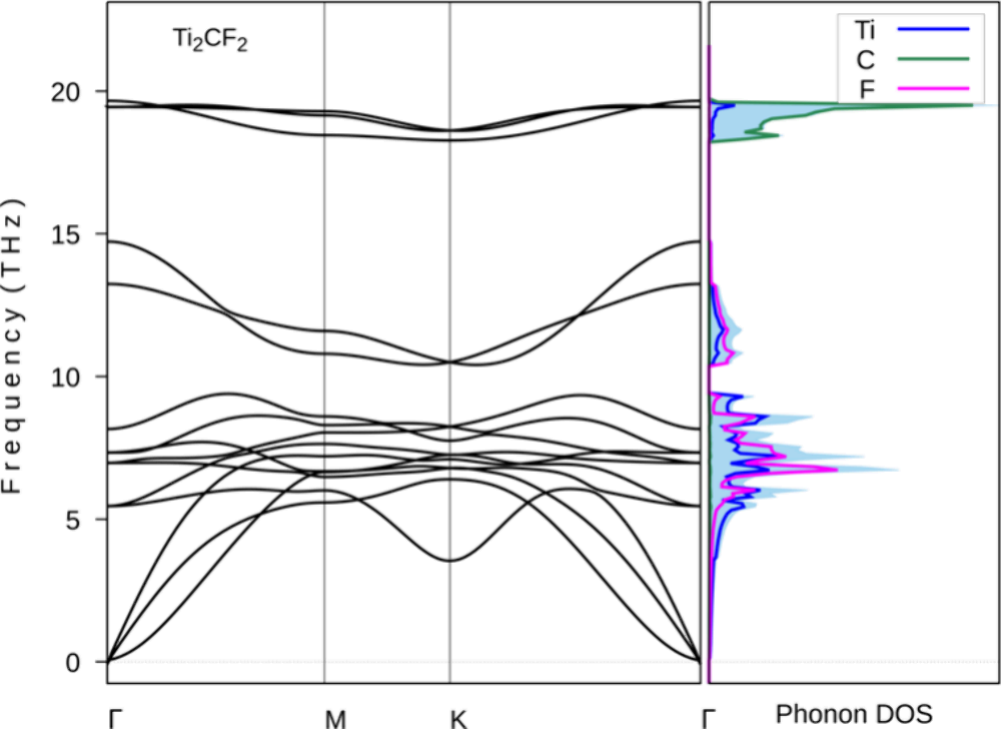
Phonon dispersion curves and phonon DOS for the Ti_2_CF_2_ MXenes.

Following these stability considerations, the electronic band structure of the Ti_2_CF_2_ MXene is determined, as illustrated in Figure 3, alongside the partial density of states (PDOS). As demonstrated in the figure, there is an absence of a gap between the valence and conduction bands. Consequently, this material is categorized as a metal. The PDOS plot indicates that the d states of Ti atoms contribute more significantly to the overall PDOS.

**Figure 3:**
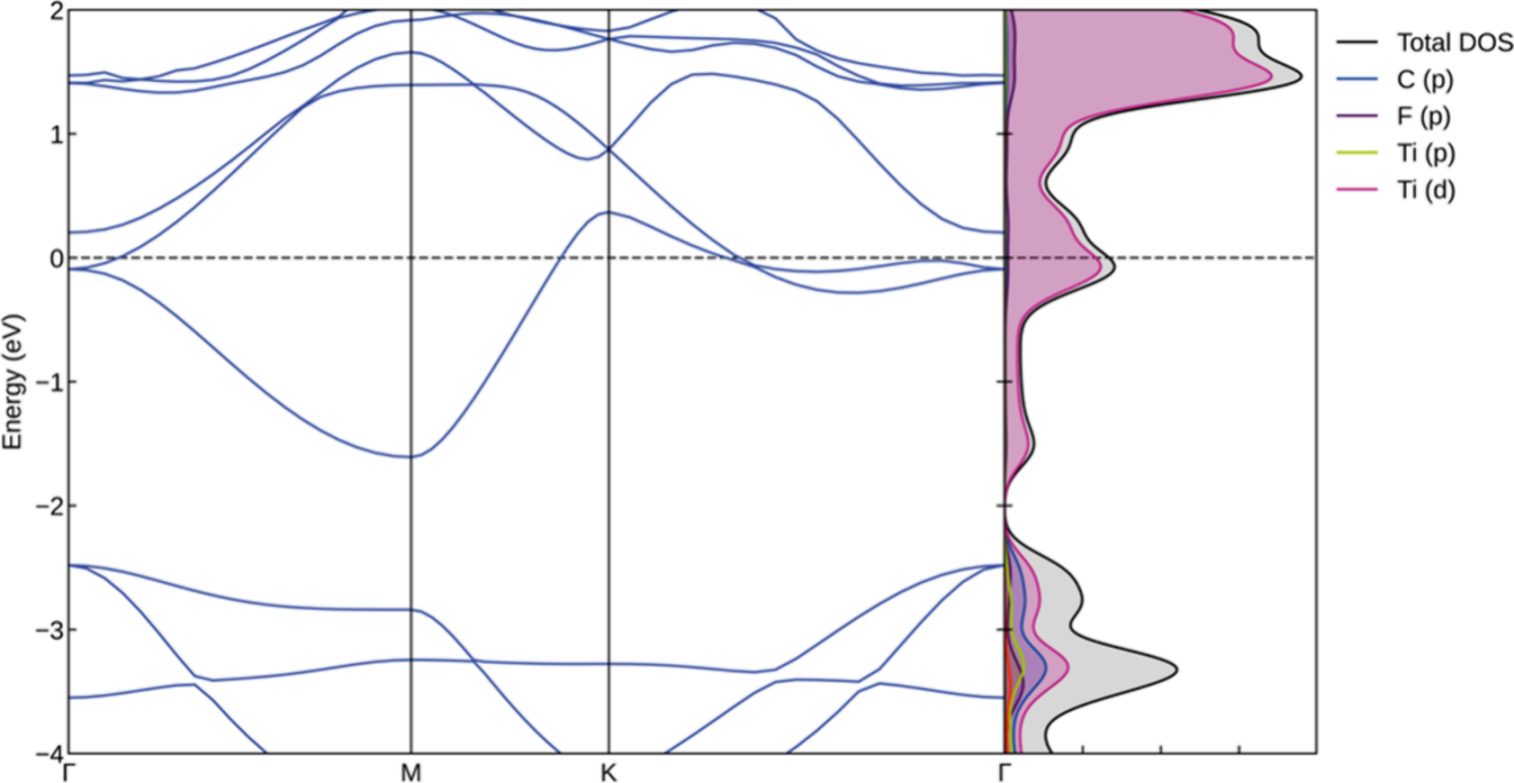
Electronic band structure and DOS plots for the Ti_2_CF_2_ MXenes.

### Li Decoration for Ti_2_CF_2_ MXene

Metal decoration is a proven strategy for enhancing the hydrogen storage capacity of two-dimensional materials. Therefore, lithium was selected to functionalize the Ti_2_CF_2_ monolayer. As illustrated in Figure 4, three distinct adsorption sites were investigated: Li_*x*_ (atop the F atom), Li_*y*_ (atop the Ti atom), and Li_*z*_ (atop the C atom). Following geometric optimization, the binding energies were calculated using an equation^54^ and are summarized in Table 2 alongside the corresponding bond lengths. The results demonstrate that the Li_*z*_ configuration exhibits the most favorable binding energy of −1.17 eV and the shortest vertical separation. This confirms it as the most energetically stable position. Consequently, the Li_*z*_ site was selected as the basis for further investigation. Building on this stability, the study was extended to a double-sided decoration strategy as shown in Figure 5. Structural optimization of this double-sided system confirmed its thermodynamic stability with a binding energy of −1.14 eV/atom. This value is comparable to the one-sided case. The equilibrium distance between the Li atom and the surface is 0.95 Å on both sides. Accordingly, this double-sided configuration was adopted for detailed hydrogen storage analysis.

**Figure 4:**
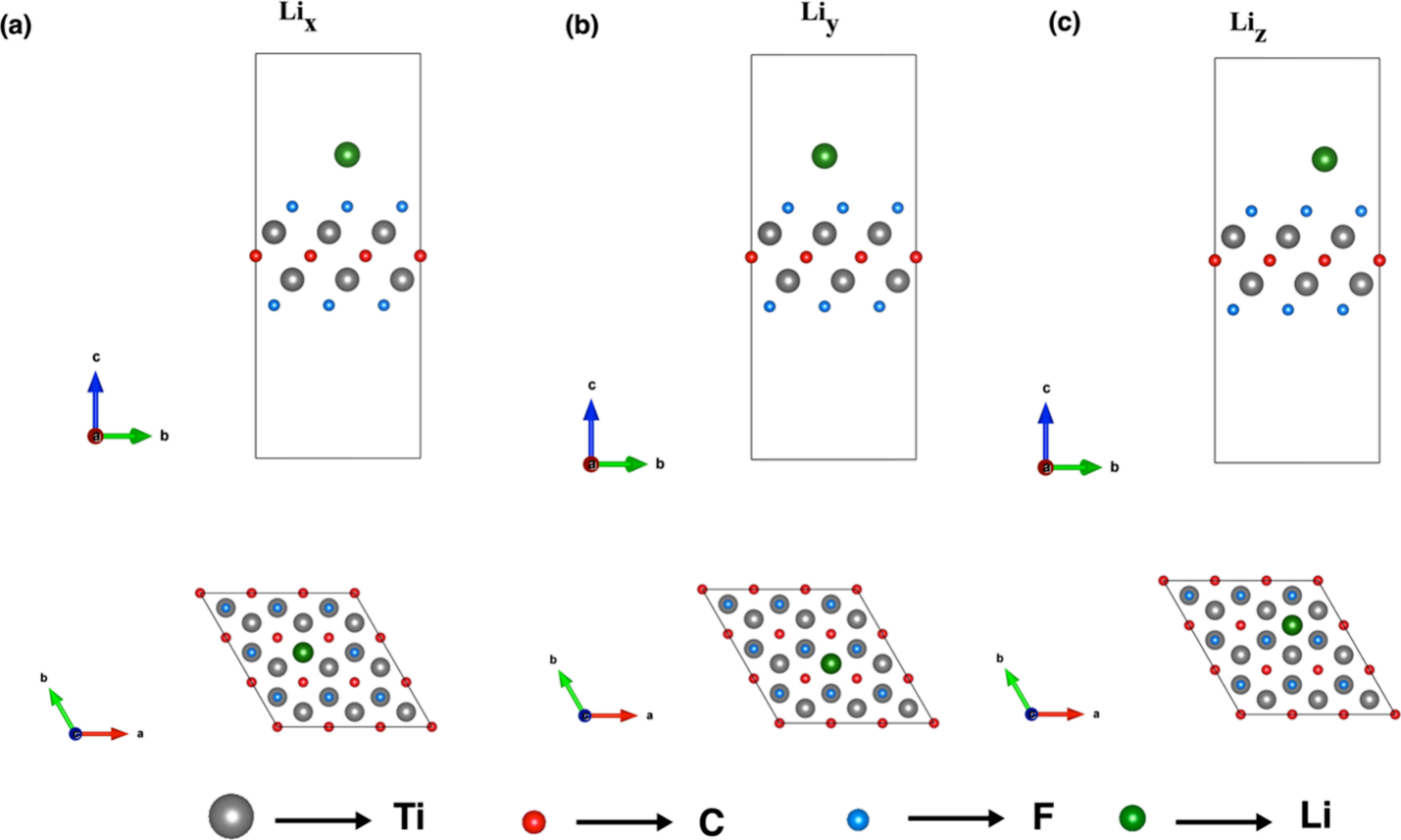
Side and top views of (a) Li_*x*_, (b) Li_*y*_, and (c) Li_*z*_ decoration positions (Li atoms are in green color).

**Table 2:** Binding Energy (*E*_bind_ in eV/Atom) and the Distance between the Li Atom and the Ti_2_CF_2_ MXene (*d* in Å)

**decoration**	***E***_**bind**_	***d***
Li_*x*_	–0.47	1.69
Li_*y*_	–1.04	1.08
Li_*z*_	–1.17	1.00

**Figure 5:**
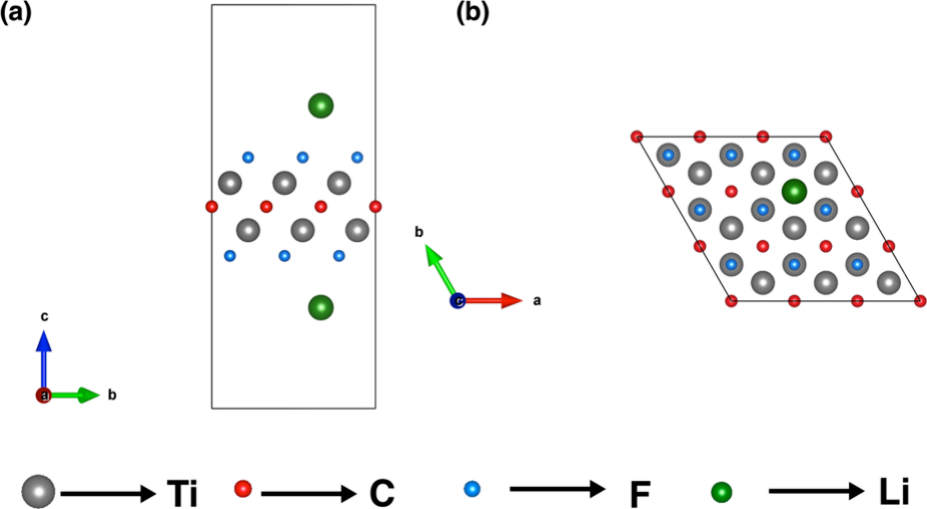
(a) Side and (b) top views of double-sided Li_*z*_ decoration.

It is worth noting that the calculated binding energies (−1.17 eV for one-sided and −1.14 eV for double-sided) of the calculated Li_*z*_ decorations appear to be slightly lower than the cohesive energy of bulk Li (approximately −1.63 eV^60^). Theoretically, this may raise a concern about the potential aggregation of Li atoms rather than the uniform distribution of Li atoms on the surface. However, the significant charge transfer from Li to the surface (as discussed in the Bader charge analysis section, Li^+^–Li^+^ between the positively charged Li ions) generates a strong Coulombic repulsion, which prevents clumping. To rigorously verify this hypothesis and ensure the structural integrity of the adsorption under operating conditions, AIMD simulations were performed at 300 K using the MLFF method. The systems were equilibrated by heating from 0 to 300 K over 10 ps (with a 1 fs time step) using a Langevin thermostat. First, the reliability of the MLFF model was confirmed; as shown in Figures S1 and S2 (Supporting Information), both the Bayesian error and the root-mean-square error (RMSE) of forces are remarkably low, indicating a well-trained model. The variations of total energy and temperature over the simulation time for one-sided and double-sided Li_*z*_ decoration are presented in Figure 6 and Figure 7, respectively. As the figures demonstrate, the total energy and temperature fluctuate within a limited and stable range. Furthermore, the snapshots of the final crystal configurations (insets in Figure 6 and Figure 7) reveal no substantial structural deformations or bond breakages. The Li atoms remain dispersed on the Ti_2_CF_2_ surface without aggregation or desorption, confirming that both one- and double-sided Li-decorated systems are dynamically stable at room temperature.

**Figure 6:**
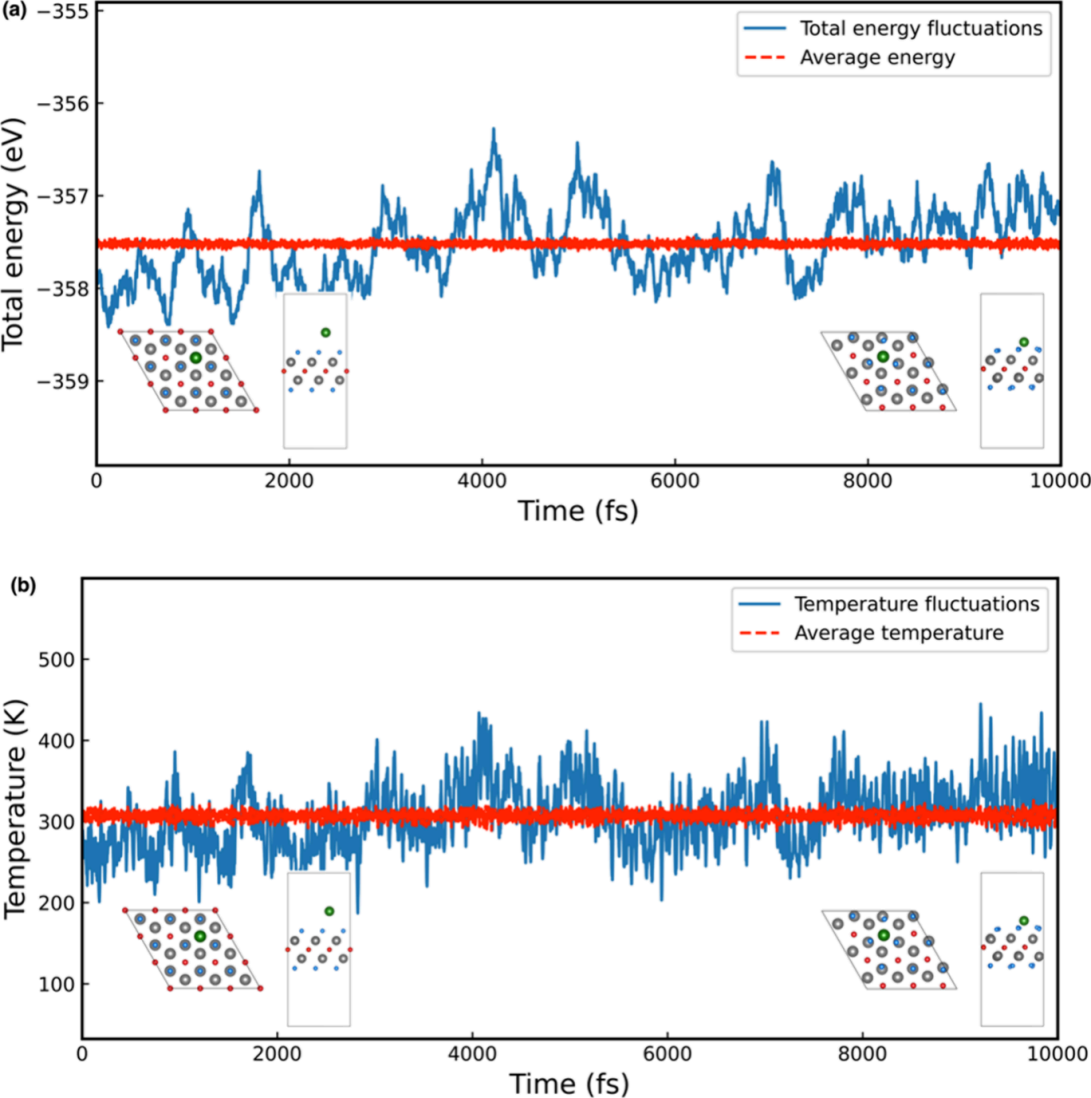
(a) Total energy and (b) temperature variation over 10,000 fs during AIMD calculations for the one-sided Li_*z*_ decoration.

**Figure 7:**
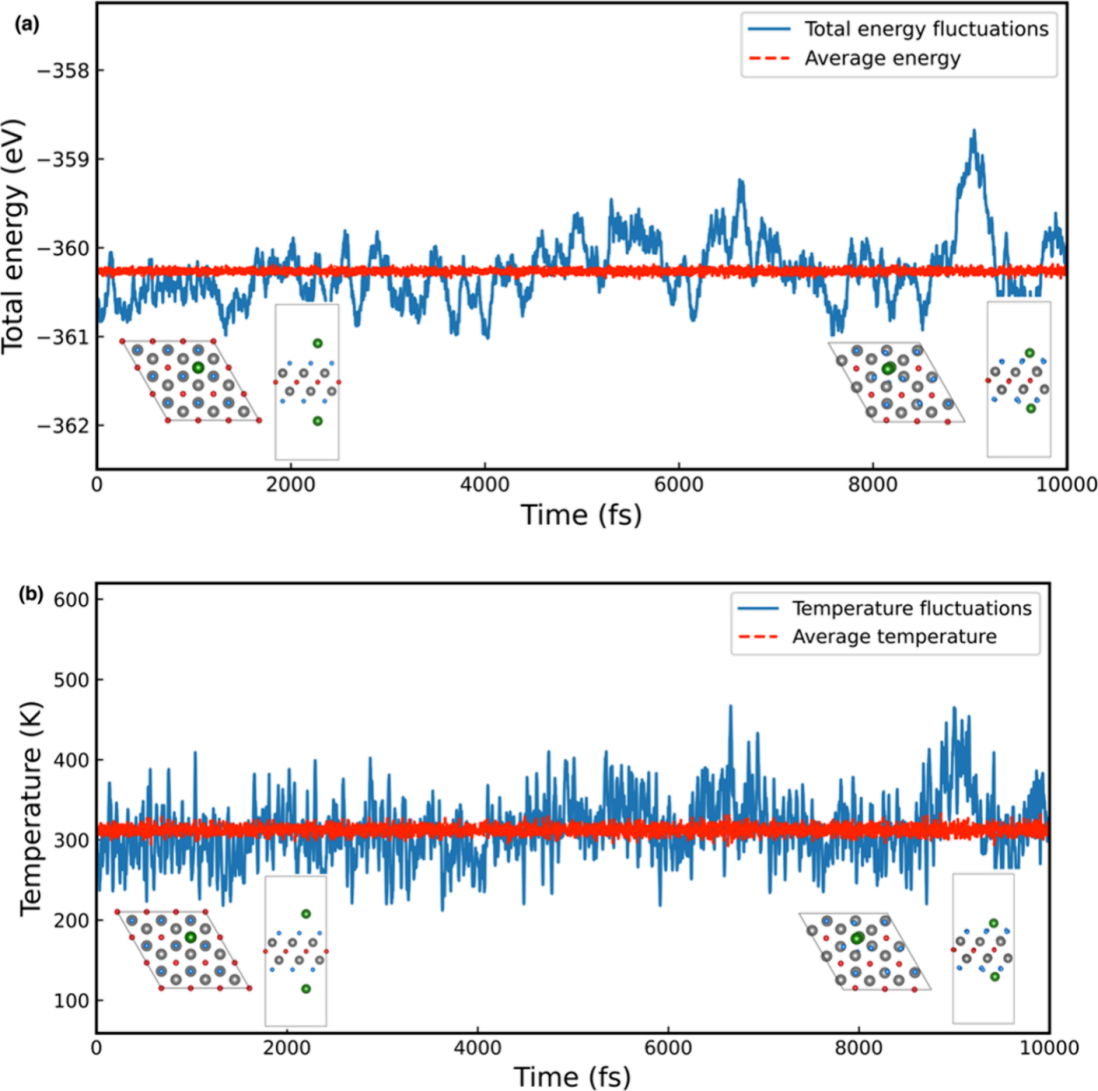
(a) Total energy and (b) temperature variation over 10,000 fs during AIMD calculations for the double-sided Li_*z*_ decoration

The electronic properties of the Li_*z*_-decorated and double-sided Li_*z*_-decorated Ti_2_CF_2_ MXenes are the focus of further investigation, along with a charge density difference analysis. The electronic band structures and PDOS plots for these systems are illustrated in Figure 8. As is evident from the figures, these systems adopt a metallic character. Moreover, the majority of contributions emanate from the d-states of Ti atoms. Figure 9 presents the charge density difference plots for the one-sided and double-sided Li_*z*_-decorated Ti_2_CF_2_ MXenes. In these images, blue regions denote areas where there is an absence of electrons, whereas yellow regions indicate regions where electrons are present in greater numbers. The phenomenon of charge transfer is driven by their binding interaction, as demonstrated by a clear charge redistribution at the interface between the Li atoms and the Ti_2_CF_2_ MXenes. Specifically, electrons are found on the surface fluorine atoms of Ti_2_CF_2_ MXenes, while there are fewer electrons around the Li atoms. This finding suggests that Li is able to donate electrons to the surface and that the Ti_2_CF_2_ MXene is capable of accepting them. The quantification of these effects necessitated the implementation of a Bader charge analysis. The findings reveal a net charge transfer of approximately |0.90|e in the one-sided configuration and |1.79|e in the double-sided case. This indicates that the Li atoms exist in a cationic state (Li^+^). Crucially, this strong ionization is vital for preventing metal clustering. Although the calculated binding energies of Li on the one-sided (−1.17 eV/atom) and double-sided (−1.14 eV/atom) cases are lower in magnitude than the cohesive energy of bulk lithium (−1.63 eV^60^), the strong electrostatic repulsion between the positively charged Li^+^ ions on the surface creates a barrier against aggregation, thereby stabilizing the dispersed decoration. The charge density difference plots (Figure 9) visually confirm this accumulation of electrons on the surface fluorine atoms and depletion around the Li sites. This cationic nature of the Li decoration is the primary driver for hydrogen adsorption, facilitating polarization-induced binding of H_2_ molecules without dissociation.

**Figure 8:**
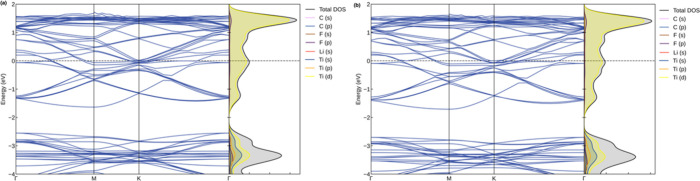
Electronic band structure and DOS plots for the (a) Li_*z*_-decorated and (b) double-sided Li_*z*_-decorated Ti_2_CF_2_ MXenes.

**Figure 9:**
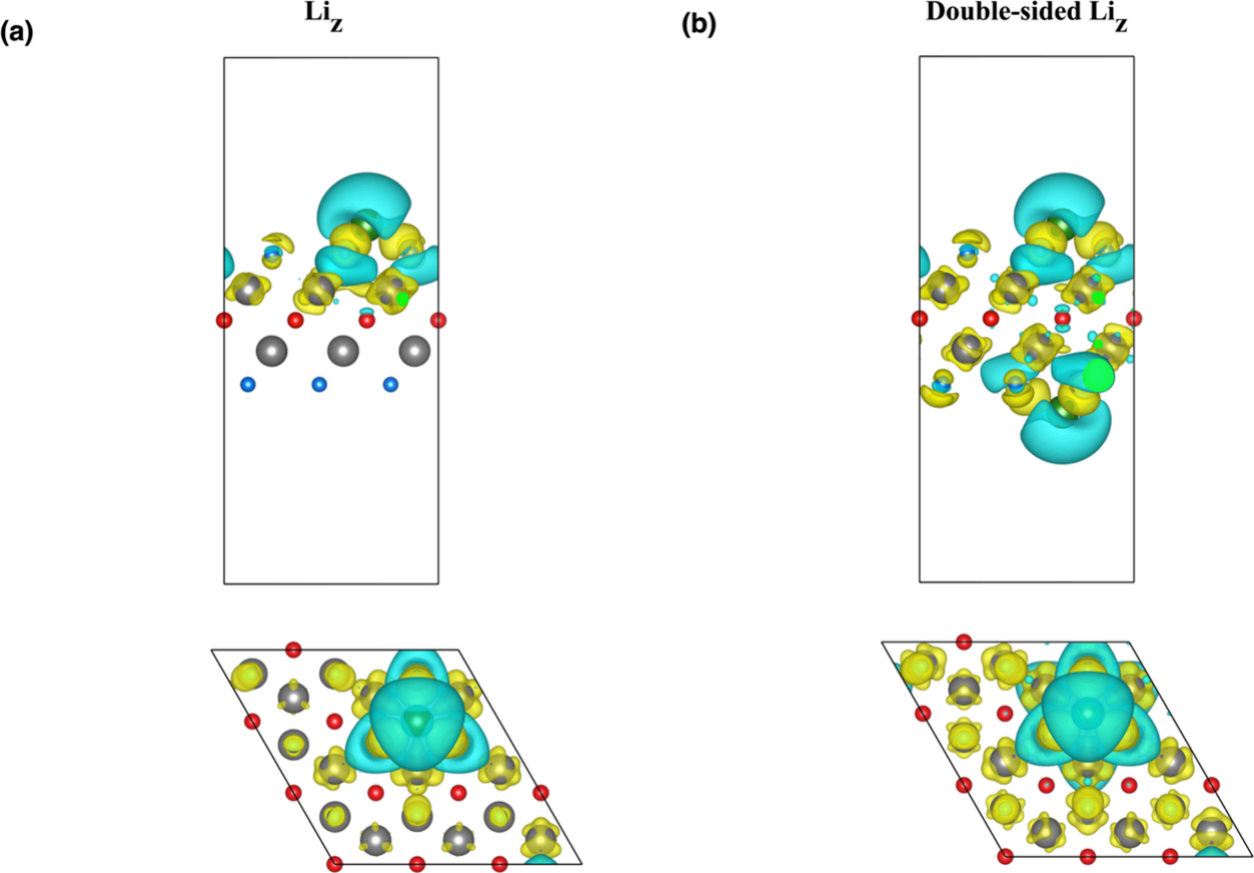
Charge density difference visualizations for the (a) Li_*z*_-decorated and (b) double-sided Li_*z*_-decorated Ti_2_CF_2_ MXenes for side and top views (isosurface level = 0.0013 e/Å^3^).

### Hydrogen Storage Studies for the Li-Decorated Ti_2_CF_2_ MXene

The hydrogen molecules are adsorbed on the Li-decorated Ti_2_CF_2_ MXene for both one-sided and double-sided decorations in order to determine the hydrogen storage capacities of these systems. The hydrogen molecules' positions are determined using the CLICH algorithm. Figure 10 shows the hydrogen positions for one-sided decoration for a 2 × 2 × 1 boundary. As the number of hydrogen molecules increases, the cap is not well seen; therefore, a 2 × 2 × 1 boundary allows to visualize the hydrogen cap clearly. For the one-sided decoration case, the number of hydrogen molecules increases up to 15 H_2_. The hydrogen positions for the double-sided decoration are shown in Figure 11. For the double-sided case, the visualizations are taken from the perspective of the system in order to show both upper and lower hydrogen positions. For this case, the number of hydrogen molecules increases up to 30 H_2_. The adsorption energy is crucial whether these hydrogen molecules adsorbed on these systems or not. The adsorption energies are listed in Tables 3 and 4, which are calculated using the equation given in ref 54. The adsorption energy becomes negative for 14 H_2_ and 15 H_2_ for one-sided decoration, while it is for 28 H_2_ and 30 H_2_ for the double-sided case. This means that for these H_2_ adsorptions, the hydrogen molecules are not adsorbed on the Li-decorated Ti_2_CF_2_ MXenes. The adsorption energy is also used to consider when assessing hydrogen storage applications, with a range of 0.15–0.60 eV/H_2_ deemed sufficient for practical hydrogen storage systems.^61–63^ This adsorption energy range is set by the U.S. Department of Energy (DOE),^64^ and according to the tables, the one-sided decoration has suitable adsorption energies for 7 H_2_ to 12 H_2_, while the double-sided decoration has suitable adsorption energies for 14 H_2_ to 24 H_2_ according to the tables.^65^ Tables 3 and 4 also list the minimum and maximum distance between the Li atom and H atoms and the hydrogen bond lengths. The distance between the Li atom and H atoms increases as the number of adsorbed hydrogen molecules increases for both one-sided and double-sided decorations. Also, the bond length for H_2_ molecules slightly decreases as the number of adsorbed hydrogen molecules increases for both one-sided and double-sided decorations.

**Figure 10:**
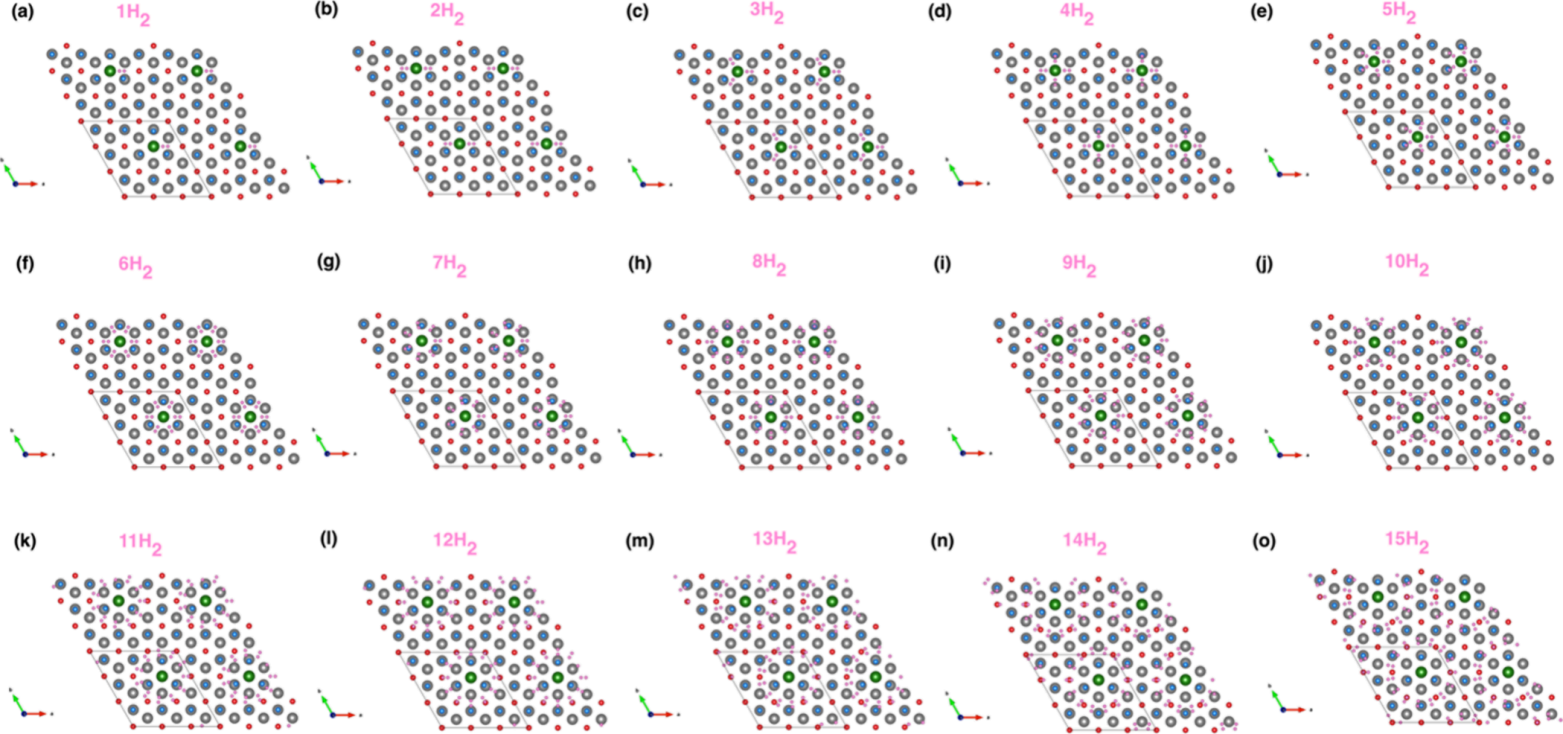
Optimized hydrogen positions for (a) 1H_2_/Li_*z*_-Ti_2_CF_2_, (b) 2H_2_/Li_*z*_-Ti_2_CF_2_, (c) 3H_2_/Li_*z*_-Ti_2_CF_2_, (d) 4H_2_/Li_*z*_-Ti_2_CF_2_, (e) 5H_2_/Li_*z*_-Ti_2_CF_2_, (f) 6H_2_/Li_*z*_-Ti_2_CF_2_, (g) 7H_2_/Li_*z*_-Ti_2_CF_2_, (h) 8H_2_/Li_*z*_-Ti_2_CF_2_, (i) 9H_2_/Li_*z*_-Ti_2_CF_2_, (j) 10H_2_/Li_*z*_-Ti_2_CF_2_, (k) 11H_2_/Li_*z*_-Ti_2_CF_2_, (l) 12H_2_/Li_*z*_-Ti_2_CF_2_, (m) 13H_2_/Li_*z*_-Ti_2_CF_2_, (n) 14H_2_/Li_*z*_-Ti_2_CF_2_, and (o) 15H_2_/Li_*z*_-Ti_2_CF_2_ systems (H atoms are in pink color.)

**Figure 11:**
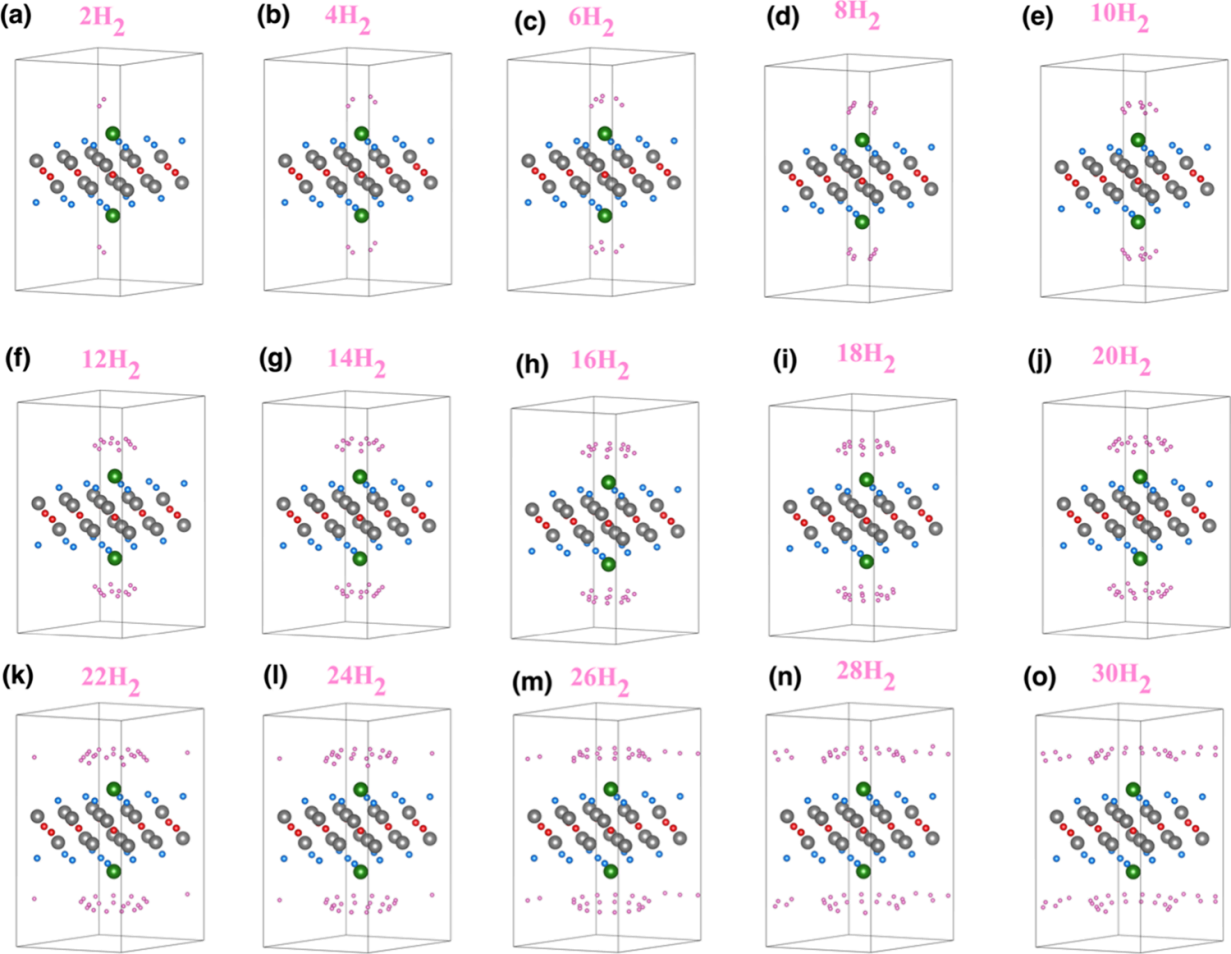
Optimized hydrogen positions for (a) 2H_2_/double-sided Li_*z*_-Ti_2_CF_2_, (b) 4H_2_/double-sided Li_*z*_-Ti_2_CF_2_, (c) 6H_2_/double-sided Li_*z*_-Ti_2_CF_2_, (d) 8H_2_/double-sided Li_*z*_-Ti_2_CF_2_, (e) 10H_2_/double-sided Li_*z*_-Ti_2_CF_2_, (f) 12H_2_/double-sided Li_*z*_-Ti_2_CF_2_, (g) 14H_2_/double-sided Li_*z*_-Ti_2_CF_2_, (h) 16H_2_/double-sided Li_*z*_-Ti_2_CF_2_, (i) 18H_2_/double-sided Li_*z*_-Ti_2_CF_2_, (j) 20H_2_/double-sided Li_*z*_-Ti_2_CF_2_, (k) 22H_2_/double-sided Li_*z*_-Ti_2_CF_2_, (l) 24H_2_/double-sided Li_*z*_-Ti_2_CF_2_, (m) 26H_2_/double-sided Li_*z*_-Ti_2_CF_2_, (n) 28H_2_/double-sided Li_*z*_-Ti_2_CF_2_, and (o) 30H_2_/double-sided Li_*z*_-Ti_2_CF_2_ systems

**Table 3:** Adsorption Energy (*E*_ads_ in eV), the Distance between a H Atom and the Li Atom (*d*_H–Li_ in Å), and the Average Hydrogen Bond Length (*d*_H–H_ in Å) for *n*H_2_/Li_*z*_-Ti_2_CF_2_ Systems

		***d***_**H–Li**_		
***n*H**_**2**_	***E*_ads_**	min	max	*d*_H–H_
1H_2_	0.79	2.90	3.10	0.74
2H_2_	0.79	3.00	3.09	0.74
3H_2_	0.78	3.09	3.26	0.74
4H_2_	0.74	3.18	3.39	0.74
5H_2_	0.75	3.41	3.61	0.74
6H_2_	0.70	3.58	3.72	0.73
7H_2_	0.65	3.74	3.86	0.73
8H_2_	0.71	4.25	4.30	0.73
9H_2_	0.61	4.21	4.25	0.72
10H_2_	0.47	4.29	4.30	0.72
11H_2_	0.24	4.35	4.34	0.72
12H_2_	0.27	4.61	4.66	0.72
13H_2_	0.12	4.65	4.88	0.73
14H_2_	–0.16	4.73	5.02	0.71
15H_2_	–0.39	4.41	5.46	0.71

**Table 4:** Adsorption Energy (*E*_ads_ in eV), the Distance between a H Atom and a Li Atom (*d*_H–Li_ in Å), and the Average Hydrogen Bond Length (*d*_H–H_ in Å) for *n*H_2_/Double-Sided Li_*z*_-Ti_2_CF_2_ Systems

		***d***_**H–Li**_		
***n*H**_**2**_	***E*_ads_**	**min**	**max**	*d*_H–H_
2H_2_	0.79	2.91	3.04	0.74
4H_2_	0.78	3.03	3.13	0.74
6H_2_	0.78	3.14	3.32	0.74
8H_2_	0.74	3.21	3.44	0.74
10H_2_	0.74	3.40	3.62	0.74
12H_2_	0.73	3.65	3.81	0.74
14H_2_	0.61	3.71	3.84	0.73
16H_2_	0.63	3.98	4.08	0.73
18H_2_	0.55	4.15	4.21	0.73
20H_2_	0.62	4.36	4.73	0.73
22H_2_	0.43	4.55	4.57	0.72
24H_2_	0.31	4.67	4.77	0.72
26H_2_	0.13	4.74	5.00	0.72
28H_2_	–0.09	4.75	5.23	0.72
30H_2_	–0.17	4.95	5.35	0.71

A comprehensive study of the electronic properties of Li-decorated Ti_2_CF_2_ MXenes materials has been conducted. All systems exhibited metallic properties, and the results for 13H_2_/Li_*z*_-Ti_2_CF_2_ and 26H_2_/double-sided Li_*z*_-Ti_2_CF_2_ systems are shown in Figure 12. The figure also includes detailed PDOS curves. As illustrated in Figure 12, the predominant contribution to the PDOS originates from the d orbitals of the Ti atom. Furthermore, the contribution of the s orbital of the H atom is observed in the range of −2 to −3 eV. The charge density difference plots for the 13H_2_/Li_*z*_-Ti_2_CF_2_ and 26H_2_/double-sided Li_*z*_-Ti_2_CF_2_ systems are shown in Figure 13. As illustrated in Figure 13, a discernible charge depletion is evident around the Li decoration, accompanied by charge accumulation surrounding the adsorbed H_2_ molecules. This phenomenon is indicative of electron transfer from Li to H_2_ molecules and, consequently, polarization of the H–H bond without dissociation. In the single-sided case, the charge distribution is localized, consistent with moderate and reversible physical adsorption of H_2_. In the double-sided configuration, the charge distribution becomes more pronounced and continuous due to higher H_2_ adsorption, but H_2_ molecules remain intact in this system, confirming that the interaction remains in the desired polarization-focused physical adsorption regime. It has been demonstrated that the combination of charge density difference plots and Bader charge analysis provides a comprehensive and consistent picture of the hydrogen adsorption mechanism on the Li-decorated Ti_2_CF_2_ surface. The Bader charges of Li atoms have been determined to be +0.90|e| and +1.79|e| for one-sided and double-sided cases, respectively. This finding is in agreement with the observed charge density difference plots. The Ti atoms exhibit a charge depletion phenomenon, characterized by the presence of +27.68|e| and +27.35|e| for one-sided and double-sided cases, respectively. It has been established that C and F atoms function as charge acceptors, with Bader charges of −15.24|e| and −13.34|e| for one-sided cases and −15.35|e| and −13.78|e| for double-sided cases, respectively. Moreover, it has been demonstrated that hydrogen molecules exhibit minimal net charge transfer, with values of −0.10|e| and +0.10|e| for the one-sided case and −0.22|e| and +0.21|e| for the double-sided case. This suggests that hydrogen molecules predominantly interact via physisorption.

**Figure 12:**
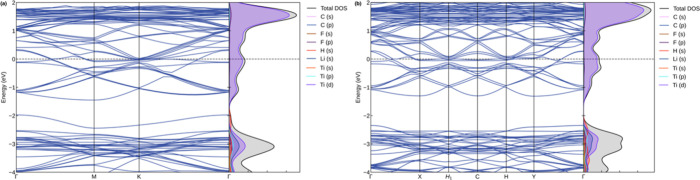
Electronic band structure and DOS plots for the (a) 13H_2_/Li_*z*_-Ti_2_CF_2_ and (b) 26H_2_/double-sided Li_*z*_-Ti_2_CF_2_ systems.

**Figure 13:**
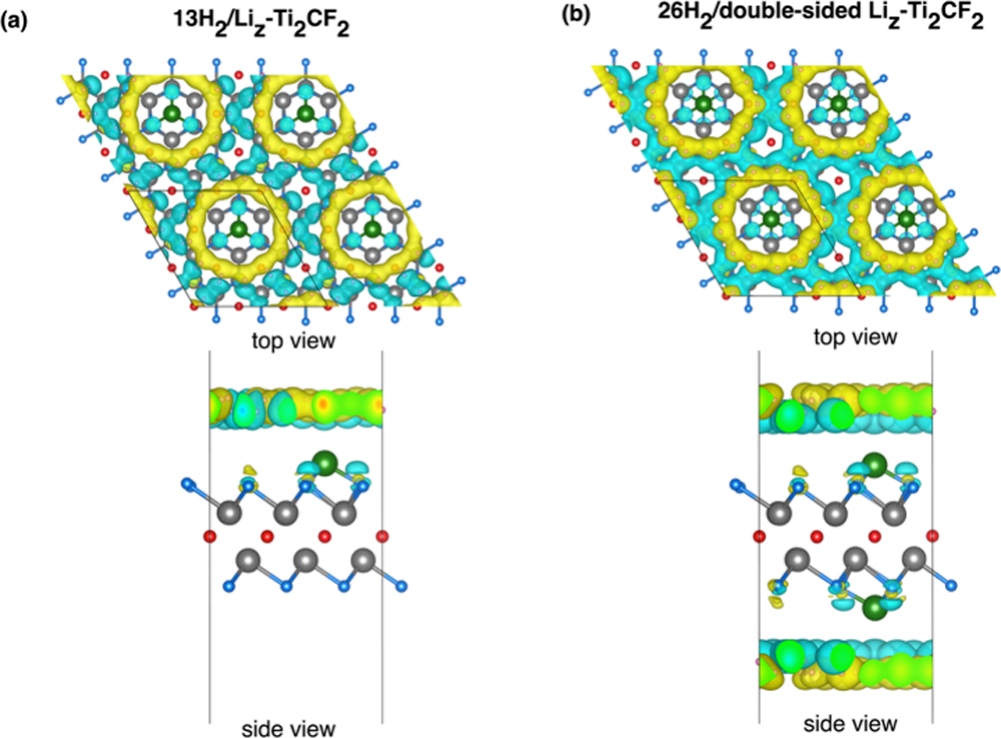
Charge density difference visualizations for the (a) 13H_2_/Li_*z*_-Ti_2_CF_2_ and (b) 26H_2_/double-sided Li_*z*_-Ti_2_CF_2_ systems (isosurface level = 0.00008 e/Å^3^).

Within the domain of research focused on the subject of hydrogen storage, the gravimetric assessment of hydrogen storage capacity represents a pivotal component of research endeavors.

The gravimetric hydrogen storage capacity (*C*_wt_ %) is a decisive parameter for evaluating the practical applicability of hydrogen storage materials. The U.S. Department of Energy (DOE) has established a technical target of 5.5 wt % for light-duty vehicles by the year 2025.^65^ To evaluate the performance of the studied systems, the gravimetric capacity was calculated using eq 1:^66,67^

$$C_{\mathrm{wt}}\;\%=\frac{n_{H_{2}}m_{H_{2}}}{(n_{H_{2}}m_{H_{2}})+m_{n{\mathrm{Li}‐\mathrm{Ti}}_{2}{\mathrm{CF}}_{2}}}\times 100$$ 1

where $n_{H_{2}},\;m_{H_{2}},\;\mathrm{and}\;m_{n{\mathrm{Li}‐\mathrm{Ti}}_{2}{\mathrm{CF}}_{2}}$ are the number of adsorbed hydrogen molecules, the mass of a hydrogen molecule, and the mass of the Li-decorated Ti_2_CF_2_ system, respectively.

The calculated hydrogen storage capacities, summarized in Tables 5 and 6, demonstrate that the double-sided Li decoration strategy significantly enhances performance, effectively doubling the maximum gravimetric capacity from 1.95 wt % (one-sided, 13 H_2_) to 3.81 wt % (26 H_2_). Although this value falls short of the stringent 2025 DOE system target of 5.5 wt %, the Ti_2_CF_2_ system remains highly competitive when compared to the DFT studies on similar 2D substrates. For instance, while it exhibits a lower theoretical capacity than lightweight carbon-based frameworks like Li-decorated graphyne (up to 18.6 wt %^68^), Ti_2_CF_2_ offers superior experimental stability and mechanical robustness. Moreover, it outperforms isostructural heavy-metal MXenes such as Hf_2_CF_2_ ^54^ due to the lighter atomic mass of titanium and provides a comparable capacity window to Y-decorated MoS_2_ (4.56 wt %^69^) without the complex electronic hybridization issues. These comparisons suggest that while Li-decorated Ti_2_CF_2_ is a promising and stable candidate, further structural engineering such as constructing van der Waals heterostructures or increasing the surface area may be required to fully satisfy commercial onboard storage targets.

**Table 5:** Gravimetric Hydrogen Storage Capacities (*C*_wt_ %) and Hydrogen Desorption Temperature (*T*_des_ in K) for *n*H_2_/Li_*z*_-Ti_2_CF_2_ Systems

*n*H_2_	*C*_wt_	*T*_des_
1H_2_	0.15	588.92
2H_2_	0.31	587.00
3H_2_	0.46	577.87
4H_2_	0.61	548.23
5H_2_	0.76	555.53
6H_2_	0.91	520.43
7H_2_	1.06	482.78
8H_2_	1.21	529.79
9H_2_	1.36	456.04
10H_2_	1.51	346.15
11H_2_	1.66	176.27
12H_2_	1.81	203.15
13H_2_	1.95	88.62

**Table 6:** Gravimetric Hydrogen Storage Capacities (*C*_wt_ %) and Hydrogen Desorption Temperature (*T*_des_ in K) for *n*H_2_/Double-Sided Li_*z*_-Ti_2_CF_2_ Systems

***n*H**_**2**_	***C***_**wt**_	***T***_**des**_
2H_2_	0.30	583.91
4H_2_	0.61	581.18
6H_2_	0.91	575.66
8H_2_	1.20	553.15
10H_2_	1.50	549.40
12H_2_	1.80	544.98
14H_2_	2.09	454.40
16H_2_	2.38	464.43
18H_2_	2.67	409.77
20H_2_	2.96	457.94
22H_2_	3.24	316.62
24H_2_	3.53	232.04
26H_2_	3.81	97.54

In addition to storage capacity, the operating conditions for hydrogen release are governed by the desorption temperature (*T*_des_). This was calculated using eq 2 based on the van’t Hoff relation.^70^

$$T_{\mathrm{des}}=\left(\frac{E_{\mathrm{ads}}}{K_{B}}\right){\left(\frac{\Delta S}{R}-\ln(P)\right)}^{-1}$$ 2

where *E*_ads_ is the adsorption energy, *K*_B_ is the Boltzmann constant, *R* is the gas constant, *P* is the equilibrium pressure (1 atm), and Δ*S* is the entropy change of hydrogen transition from gas to solid phase (130 J mol^–1^ K^–1^).^70^

Tables 5 and 6 present the hydrogen desorption temperatures for the *n*H_2_/Li_*z*_-Ti_2_CF_2_ and *n*H_2_/double-sided Li_*z*_-Ti_2_CF_2_ systems, respectively. A comprehensive evaluation of the values presented in the aforementioned table has revealed a discernible relationship between the temperature of hydrogen release and the quantity of stored hydrogen within the *n*H_2_/Li_*z*_-Ti_2_CF_2_ and *n*H_2_/double-sided Li_*z*_-Ti_2_CF_2_ systems. It is evident that as the amount of stored hydrogen increases, the hydrogen release temperatures decrease. It has been established that the lowest recorded temperature at which hydrogen desorption occurs in the *n*H_2_/Li_*z*_-Ti_2_CF_2_ and *n*H_2_/double-sided Li_*z*_-Ti_2_CF_2_ systems is obtained for 13H_2_ and 26H_2_ cases, respectively. This indicates that at maximum capacity, the hydrogen is weakly bound, facilitating release at low temperatures, though cryogenic conditions may be required to maintain full capacity.

It is imperative to consider hydrogen’s adsorption and desorption processes at various temperatures and pressures. In order to obtain the aforementioned quantity, a thermodynamic analysis was performed using the grand canonical partition function *Z*, as given in eq 3.^71–73^

$$Z=1+\sum_{i=1}^{n}e^{-(E_{\mathrm{ads}}^{i}-\mu )/k_{B}T}$$ 3

where *n* signifies the maximum number of adsorbed H_2_ molecules and *E*_*ads*_^*i*^, μ, and *k*_B_ correspond to the adsorption energy of the *n* ^th^ adsorbed H_2_ molecule, the chemical potential of the gas phase of the H_2_ molecule, and the Boltzmann constant, respectively. μ is dependent on temperature and pressure and can be determined using eq 4.^71–73^

$$\mu =\Delta H+T\Delta S+k_{B}T\ln\frac{P}{P_{0}}$$ 4

In this equation, the enthalpy change, entropy change, pressure, and atmospheric pressure are represented as Δ*H*, Δ*S*, *P*, and *P*_0_, respectively. Δ*H* + *T*Δ*S* can be obtained from the experimental database.^74^ Equation 5 was utilized to calculate the quantity of stored H_2_ molecules, with *N*_0_ denoting the maximum number of adsorbed H_2_ molecules at 0 K.^71–73^

$$N=N_{0}\left[\frac{Z-1}{Z}\right]$$ 5

The number of adsorbed H_2_ molecules for the 13H_2_/Li_*z*_-Ti_2_CF_2_ and 26H_2_/double-sided Li_*z*_-Ti_2_CF_2_ systems is presented as a function of *P* and *T* in Figures 14 and 15, respectively. The figures demonstrate that the H_2_ molecules are adsorbed at low temperatures and elevated pressures and desorbed at high temperatures and reduced pressures, for both systems.

**Figure 14:**
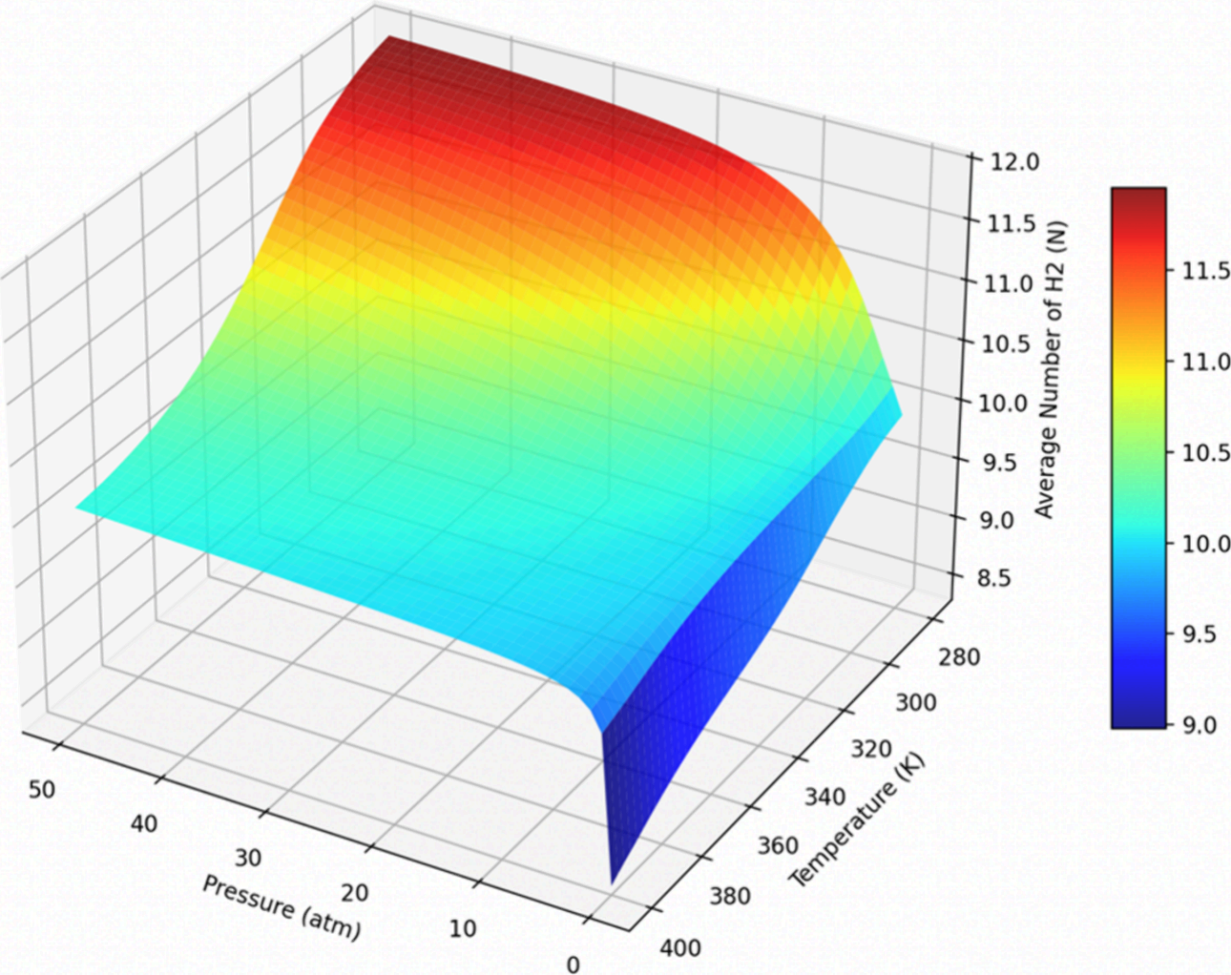
Average number of H_2_ molecules as a function of pressure and temperature for the 13H_2_/Li_*z*_-Ti_2_CF_2_ system.

**Figure 15:**
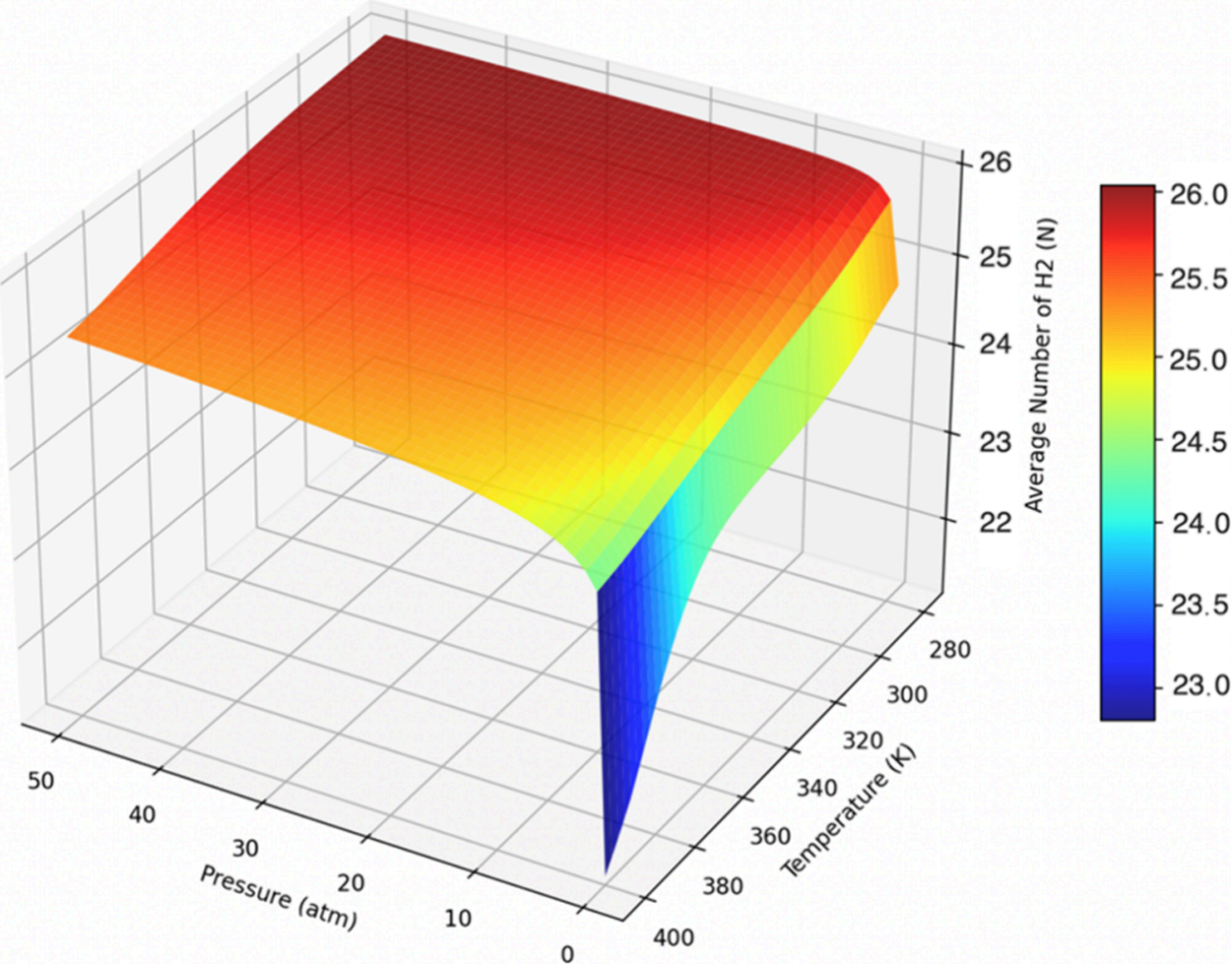
Average number of H_2_ molecules as a function of pressure and temperature for the 26H_2_/double-sided Li_*z*_-Ti_2_CF_2_ system.

To evaluate the hydrogen storage performance of the Li-decorated Ti_2_CF_2_ systems, a comprehensive comparison with previously reported storage materials is summarized in Table 7. The calculated average adsorption energies for the Li_*z*_-Ti_2_CF_2_ and double-sided Li_*z*_-Ti_2_CF_2_ systems (0.12 and 0.13 eV/H_2_, respectively) fall within the broader literature range of 0.065–1.21 eV/H_2_. Notably, these values are slightly higher than those reported for other functionalized MXenes such as Y_2_CF_2_ (0.080 eV) and Y_2_CCl_2_ (0.065 eV),^75^ suggesting a relatively more stable adsorption environment in the Ti_2_CF_2_ framework. However, they remain lower than transition metal-doped systems like Sc-doped CNR (0.95 eV) and Ti-doped CNR (1.21 eV),^76^ which typically exhibit stronger chemisorption-like interactions.

**Table 7:** Maximum Number of Adsorbed H_2_ Molecules (*n*_max_), Adsorption Energy (|*E*_ads_| in eV/H_2_ Molecule), Gravimetric Hydrogen Storage Capacity (*C*_wt_ %), and Hydrogen Desorption Temperature (*T*_des_ in K) for Some Hydrogen Storage Systems

**systems**	***n*_max_**	**|** *E*_**ads**_ **|**	***C***_**wt**_	***T***_**des**_	**reference**
Li_*z*_-Ti_2_CF_2_	13	0.12	1.95	88.62	this study
double-sided Li_*z*_-Ti_2_CF_2_	26	0.13	3.81	97.54	this study
Y_2_CF_2_	36	0.080	3.418		^75^
Y_2_CCl_2_	36	0.065	3.000		^75^
Y_2_C(OH)_2_	36	0.085	3.477		^75^
Li–Sc_3_N_2_	5	0.28	5.9	320	^80^
Na–Sc_3_N_2_	5	0.137	5.6	320	^80^
Ti_3_C_2_T_*x*_			10.47	77	^79^
Sc-doped CNR	32	0.95	6.10	358	^76^
Ti-doped CNR	30	1.21	5.49	371	^76^
Ca-doped C_3_N_2_	22	0.24	7.53	178	^78^
Mg-doped C_3_N_2_	25	0.24	9.47	141	^78^
K-doped C_3_N_2_	24	0.19	8.21	178	^78^
6Sc–C_5_N	36	0.2131	5.810		^81^
6Ti–C_5_N	36	0.2995	5.730		^81^
6V–C_5_N	36	0.1844	5.647		^81^
Li–C_9_N_4_	6	0.20	11.9		^77^
Na–C_9_N_4_	6	0.19	8.7		^77^
K–C_9_N_4_	7	0.17	8.1		^77^

Regarding storage capacity, the double-sided Li_*z*_-Ti_2_CF_2_ system achieves a gravimetric capacity of 3.81 wt %. While this is lower than high-capacity benchmarks like Li-C_9_N_4_ (11.9 wt %)^77^ or Mg-doped C_3_N_2_ (9.47 wt %),^78^ it is superior to several MXene-based counterparts, including Y_2_CCl_2_ (3.00 wt %) and Y_2_CF_2_ (3.418 wt %).^75^ This highlights that Li decoration on the Ti_2_CF_2_ surface effectively enhances the storage density compared to some heavier transition metal MXene systems.

Finally, the hydrogen release characteristics were assessed via the desorption temperature (*T*_des_). The literature values for *T*_des_ vary significantly, ranging from 77 K (for Ti_3_C_2_T_*x*_ ^79^) to 371 K (for Ti-doped CNR^76^). The *T*_des_ obtained in this study (97.54 K) is comparable to the cryogenic release temperatures observed in Ti_3_C_2_T_*x*_, though it remains below the ambient temperature threshold (approximately 300 K) required for practical fuel cell applications. This suggests that while the Li_*z*_-Ti_2_CF_2_ system provides a stable platform for hydrogen uptake, its primary application would currently be suited for cryogenic storage conditions.

Although the present study is founded on first-principles calculations, the experimental realization of Li-decorated Ti_2_CF_2_ can be feasible through established top-down and postsynthetic methods. The precursor, Ti_2_CF_2_ MXene, can be synthesized by subjecting the Al layer to selective etching from the Ti_2_AlC MAX phase using hydrofluoric acid (HF) or a mixture of LiF and HCl, which naturally leads to surface terminations with a fluorine (−F) content. In the case of Li decoration, electrochemical intercalation or chemical lithiation can be employed.

## Conclusions

In this study, the hydrogen storage performance of Li-decorated Ti_2_CF_2_ MXene was systematically investigated using density functional theory (DFT) and ab initio molecular dynamics (AIMD) simulations. The structural stability analysis confirmed that the Ti_2_CF_2_ monolayer is both thermodynamically and mechanically stable. Among the considered adsorption sites, the Li atom was found to be most energetically favorable at the hollow site above the carbon atom (Li_*z*_). Crucially, Bader charge analysis revealed a significant charge transfer from the Li atom to the substrate, leaving the Li adatoms in a cationic state (Li^+^). This strong ionization, combined with the resultant electrostatic repulsion, effectively prevents metal clustering, a finding further validated by AIMD simulations at 300 K, which showed no aggregation. The hydrogen storage capacity was evaluated for both one-sided and double-sided decoration strategies. The results demonstrated that the Li-decorated systems adsorb hydrogen molecules via a polarization-induced physisorption mechanism, with adsorption energies falling within the ideal range (0.2–0.6 eV/H_2_) for reversible storage at moderate conditions. While the one-sided decoration yielded a gravimetric capacity of 1.95 wt % (13 H_2_), the double-sided strategy significantly enhanced this value to 3.81 wt % (26 H_2_). Although this maximum capacity remains below the ultimate DOE target of 5.5 wt %, the system exhibits superior stability compared to many other theoretical 2D materials and outperforms isostructural heavy-metal MXenes. Furthermore, the desorption temperature calculations suggest that hydrogen release is feasible at manageable temperatures. Consequently, Li-decorated Ti_2_CF_2_ stands out as a promising, stable, and reversible medium for hydrogen storage, warranting further experimental verification and structural engineering to maximize its surface area. In the field of experimental research, the focus of future studies should be oriented toward the fabrication of van der Waals (vdW) heterostructures, such as Ti_2_CF_2_/graphene, with the objective of impeding the restacking of MXene layers and thereby preserving active surface sites. Furthermore, the development of porous architectures (e.g., 3D printed MXene aerogels) could result in a significant increase in the accessible surface area, thus providing a higher density of Li decoration sites and, consequently, enabling the gravimetric capacity to meet the requirements for mobile applications.

## Supplementary Material



## Notes

MLFF Bayesian error and RMSE of forces for the one-sided and double-sided Li-decorated Ti_2_CF_2_ systems (Figures S1 and S2) (PDF)
