# Supplementary material for: Li-Decorated Ti2CF2 MXene for Efficient Solid-State Hydrogen Storage
Source: ACS Omega. 2026 Apr 10;11(15):23581–94. doi: 10.1021/acsomega.6c02092 (PMC13103757; doi:10.1021/acsomega.6c02092)
Supplement: Supplementary file 1 [file ao6c02092_si_001.pdf]

Supporting Information

Li-Decorated Ti_2CF_2 MXene for Efficient Solid-State Hydrogen Storage

Bilal Gulseven[a], Gokhan Surucu[b*], Ozge Surucu[c], Aysenur Gencer[d]

[a]Graduate School of Natural and Applied Sciences, Gazi University, 06500, Ankara, Türkiye

[b]Department of Energy Systems Engineering, Faculty of Technology, Gazi University, 06500, Ankara, Türkiye

[c]Department of Energy Systems Engineering, Faculty of Engineering and Natural Sciences, Ankara Yildirim Beyazit University, 06010, Ankara, Türkiye

[d]Department of Physics, K. O. Faculty of Science, Karamanoglu Mehmetbey University, 70200, Karaman, Türkiye

*Corresponding author: gokhansurucu@gazi.edu.tr

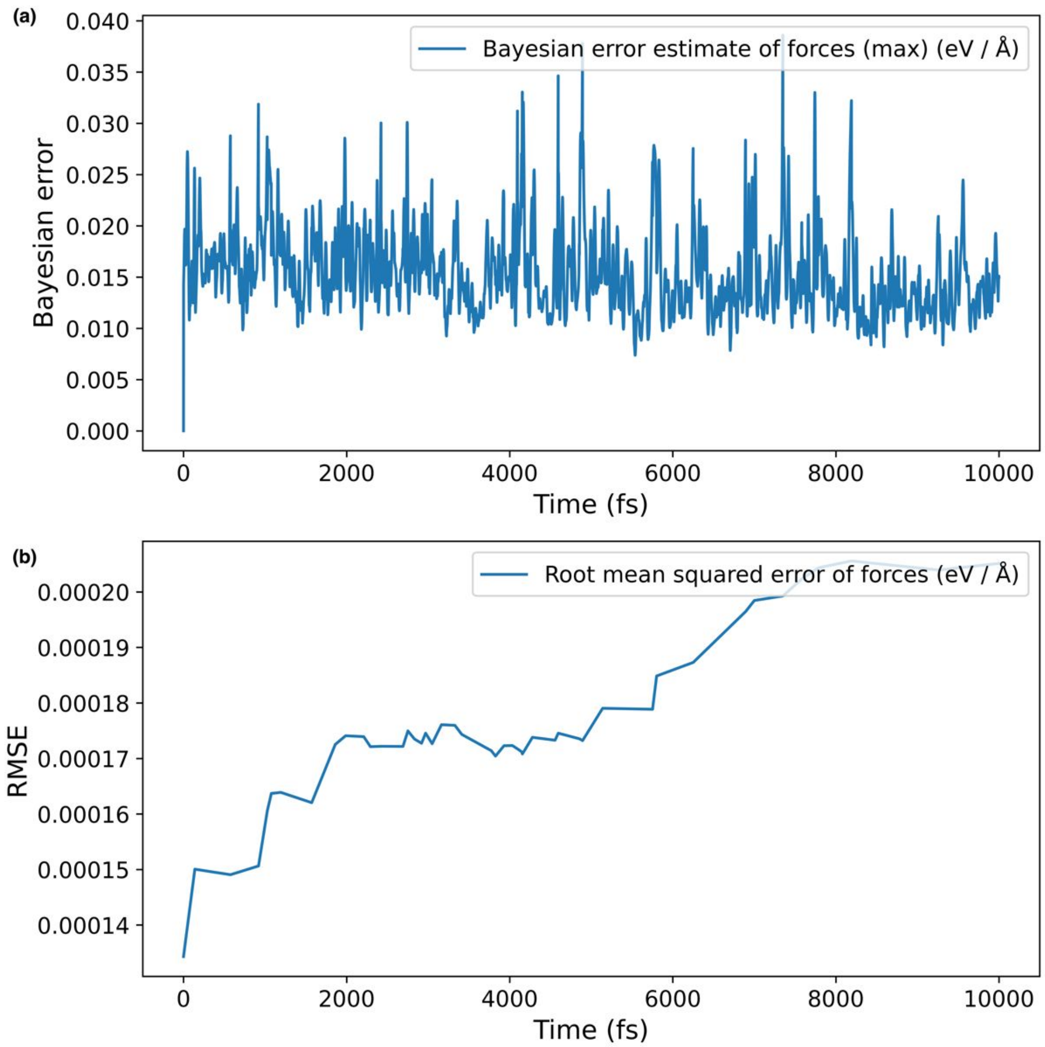

Figure S1: (a) The Bayesian error of the MLFF and (b) the root mean square error of forces for the one-sided Li_z decoration as a function of time during AIMD calculations

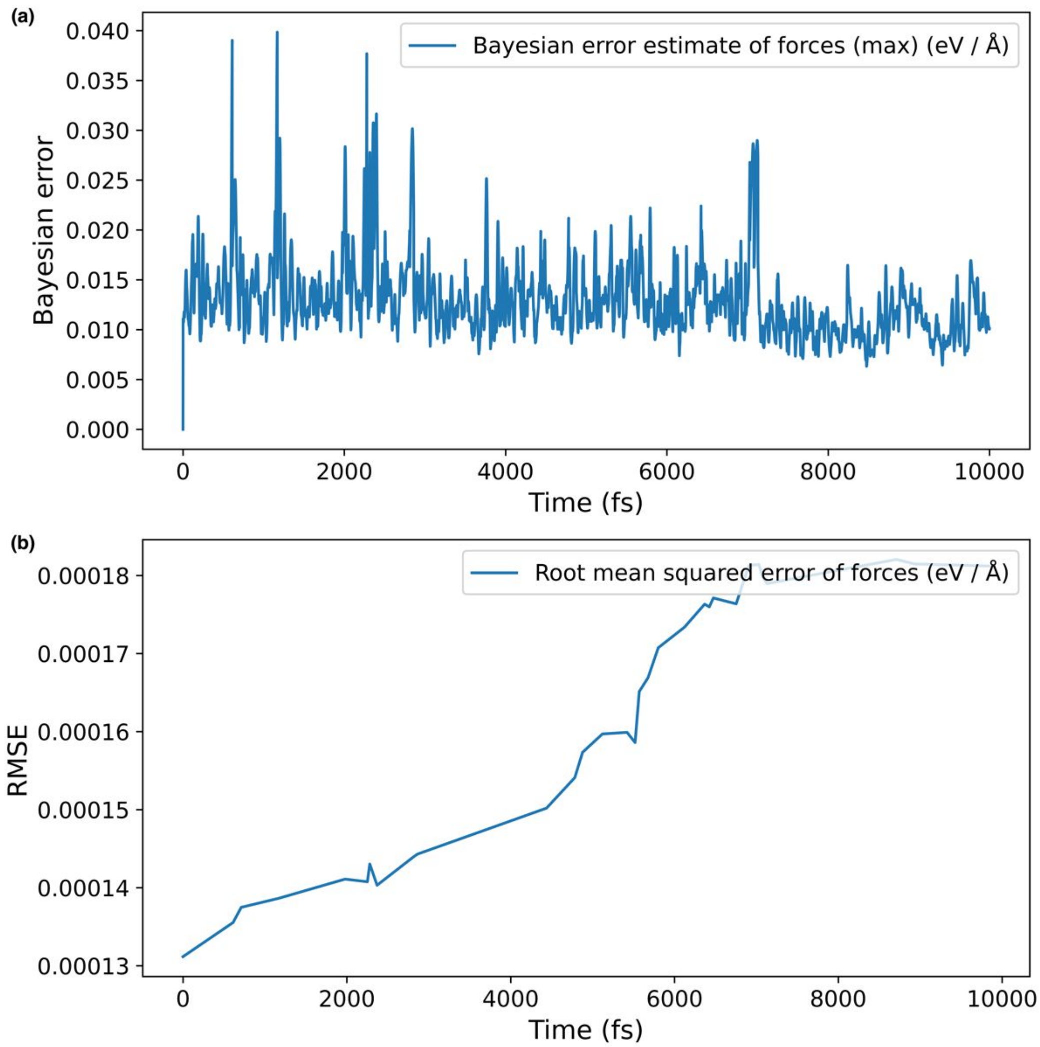

Figure S2: (a) The Bayesian error of the MLFF and (b) the root mean square error of forces for the double-sided Li_z decoration as a function of time during AIMD calculations
